# Liquid Metal-Based Electrode Array for Neural Signal Recording

**DOI:** 10.3390/bioengineering10050578

**Published:** 2023-05-10

**Authors:** Xilong Zhang, Bingxin Liu, Jingru Gao, Yiran Lang, Xiaodong Lv, Zhongshan Deng, Lin Gui, Jing Liu, Rongyu Tang, Lei Li

**Affiliations:** 1 Key Laboratory of Cryogenics, Technical Institute of Physics and Chemistry, Beijing 100190, China; 2 School of Future Technology, University of Chinese Academy of Sciences, Beijing 100049, China; 3 School of Advanced Engineering, University of Science and Technology Beijing, Beijing 100083, China; 4 School of Life Science, Beijing Institute of Technology, Beijing 100081, China; 5 Department of Biomedical Engineering, School of Medicine, Tsinghua University, Beijing 100084, China; 6 The State Key Laboratory on Integrated Optoelectronics, Institute of Semiconductors, Chinese Academy of Sciences, Beijing 100083, China

**Keywords:** liquid metal (LM), fluidic electrodes, multi-channel electrodes, neural signal recording, cortical source localization

## Abstract

Neural electrodes are core devices for research in neuroscience, neurological diseases, and neural–machine interfacing. They build a bridge between the cerebral nervous system and electronic devices. Most of the neural electrodes in use are based on rigid materials that differ significantly from biological neural tissue in flexibility and tensile properties. In this study, a liquid-metal (LM) -based 20-channel neural electrode array with a platinum metal (Pt) encapsulation material was developed by microfabrication technology. The in vitro experiments demonstrated that the electrode has stable electrical properties and excellent mechanical properties such as flexibility and bending, which allows the electrode to form conformal contact with the skull. The in vivo experiments also recorded electroencephalographic signals using the LM-based electrode from a rat under low-flow or deep anesthesia, including the auditory-evoked potentials triggered by sound stimulation. The auditory-activated cortical area was analyzed using source localization technique. These results indicate that this 20-channel LM-based neural electrode array satisfies the demands of brain signal acquisition and provides high-quality-electroencephalogram (EEG) signals that support source localization analysis.

## 1. Introduction

The neural–machine interface technique aims to build a bridge between the brain and external electronic devices that enables communication and control and achieves coordination between the human brain and machines by collecting and decoding neuro-electrical signals [1]. The development of this technique is of great significance for the diagnosis, research, and treatment of cerebral neurological diseases. As a device that connects biological nerves to electronic devices, the neural electrode is responsible for recording and modulating neural activity. Its performance directly affects neuro-electrical signals’ detection, recording, and modulation. Implantable electrodes are positioned physically closer to the neural tissue than non-invasive electrodes. They have a higher spatial resolution, enabling them to record signals with a higher signal-to-noise ratio and perform stimulation with higher spatial resolution. Their integration sensing, stimulation, and drug-delivery capabilities provide a valuable platform for health monitoring, disease diagnosis, and medical treatment [2,3,4].

In order to reduce the damage or disturbance to neural tissue, implantable electrodes should be as tiny as possible. In addition, it is required to create an implantable micro-electrode array where dozens of recording points are integrated to record signals from multiple neurons simultaneously. Many types of electrodes are manufactured using microelectromechanical systems (MEMS) technology to minimize their size and weight, minimize impairments incurred when implanted, and improve their conformity and stability. Some MEMS electrodes have been widely applied to modulate neural activity by external stimulation for the clinical treatment of neural disease [5,6]. Despite excellent electronic properties, traditional rigid probes induce a chronic inflammatory reaction because of the significant mechanical mismatch with the soft, flexible, and dynamic biological tissue [7,8,9,10]. In addition, the inflammatory response can eventually create an insulating gelatinous scar, resulting in diminished signal quality or signal gain.

To overcome this restriction, flexible electronics have become a prospective technology distinguished by their compliant mechanical performance, which includes the ability to withstand bending, twisting, and expansion [11,12,13,14]. One common strategy is to print or spray the conductive materials on a soft substrate such as polydimethylsiloxane (PDMS) [15], polyimide (PI) [16], or polyamide (PA) [17]. Silicon nanowires [18], carbon nanotubes [19], graphene [17,20], and Poly(3,4-ethylenedioxythiophene) polystyrene sulfonate (PEDOT:PSS) [21] have been used for the fabrication of brain electrodes recently. However, the electronic conductivity of these materials (for example, graphene is ~5.2 × 10^3^ S/m, and PEDOT:PSS is ~2.8 × 10^3^ S/m) is much lower than that of the metal materials (such as Pt ~9.4 × 10^6^ S/m, Ag ~6.8 × 10^7^ S/m, and Au ~4.3 × 10^7^ S/m). For a nerve electrode, electronic conductivity is an important parameter, since electrodes composed of high-conductivity materials facilitate the obtaining of high signal-to-noise ratio signals in vivo.

Liquid metals (LMs) are metallic materials that remain liquid at room temperature. Eutectic Ga–In alloy (EGaIn, 75.5% gallium and 24.5% indium) and eutectic Ga–In–Sn alloy (Galinstan, 68.5% gallium, 21.5% indium, and 10.0% tin) are the two most common liquid metals. They have excellent electronic conductivity, low melting points, and good bio-compatibility [22,23]. These Ga-based liquid metals have proven the feasibility of acute neural reconnection and stimulation [24,25,26,27,28]. Recently, the present group developed an LM-based cuff electrode and demonstrated its ability to maintain stable and effective bidirectional transmission of high signal-to-noise-ratio neural signals in freely moving rats [29]. In addition, LM nanoparticles (NPs) can also be used for the fabrication of nerve electrodes [30,31]. However, the electrical conductivity of LM NPs is much lower than that of LMs. Moreover, LMs can be directly injected into microchannels for the transfer of electrical signal, which simplifies the fabrication processing of a sensor [32,33,34].

In this study, an LM-based 20-channel neural electrode array with a Pt-encapsulation material was developed. The LM-based electrode demonstrated low interfacial impedance and excellent deformability. It had superior flexibility and tight adhesion to a rat’s skull, which facilitated the acquisition of high signal-to-noise EEG signals during implantation. The LM-based electrode, when implanted in rats, successfully recorded EEG signals under low-flow or deep anesthesia. In addition, it could record auditory-evoked potentials and localize the auditory-activated cortical area using source localization technique. 

## 2. Materials and Methods

### 2.1. Materials for Electrode Preparation

A liquid-metal alloy, EGaIn (with a composition of 75.5% gallium and 24.5% indium), was used for fabricating the electrode array, in which Gallium (99.99% purity) was bought from Shanxi Zhaofeng Gallium Company, Yangquan, Shanxi, China, and Indium (99.995% purity) was bought from Zhuzhou Keneng New Materials Company, Zhuzhou, Hunan, China. The two metals were put in a vessel with a weight ratio of 75.5 (Gallium): 24.5 (Indium) and then stirred after heating at 180 °C for 3 h to ensure that they were well blended.

The substrate was made by PDMS (mixture of base and curing agent at a ratio of 10:1 in mass, Dow Corning, Auburn, MI, USA). Platinum columns with a diameter of 0.5 mm and length of 0.5 mm were bought from Wuhu Ariter Elector-mechanical Equipment Co., Ltd., Wuhu, Anhui, China. 

### 2.2. Design and Fabrication of the LM-Based Electrode

The LM-based electrode had 20 channels. Two channels were used as reference electrodes to determine the electrode potential of the working electrode with respect to the reference electrode. Two channels serve as ground electrodes, which formed a circuit with the working electrode to allow the working electrode current to flow smoothly to ensure that the reaction under study occurred at the working electrode. This design could eliminate common-mode noise and improve the signal-to-noise ratio by differentiating the signals of different channels.

**Figure 1:**
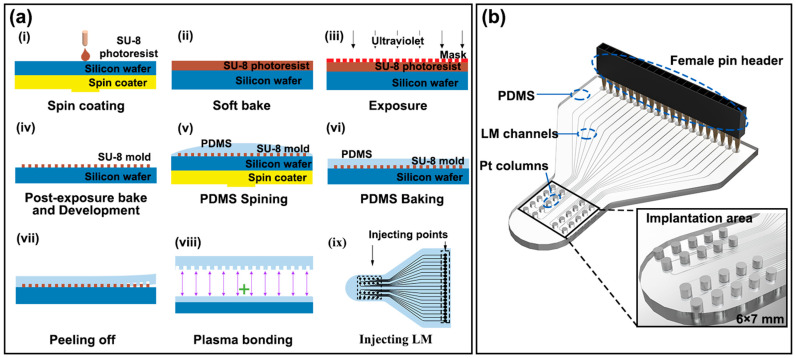
(**a**) Soft lithography process and LM-based electrode’s manufacturing operation. First, the SU-8 photoresist was coated on the silicon tray (i), and the SU-8 die was obtained on the silicon tray after pre-bake (ii), exposure (iii), post-bake and development (iv) steps. Then, PDMS was poured on the mold (v) and coated using a spin coater at 500 rpm for 2 min (vi), followed by heating at 65°C for 150 min. After that, the thin PDMS substrate was peeled off from the mold (vii,viii). After that, holes with a diameter of 0.5 mm were punched at both ends of the channel using a hole puncher (ix); (**b**) 3D rendering of the LM-based electrode’s structure and local enlargement of implant area (6 × 7 mm).

LM-based electrode channels were made using soft lithography technology. Figure 1a shows the soft lithography process and the LM-based electrode’s manufacturing operation. Firstly, SU-8 photoresist (MicroChem, SU-8 2050, Dalian, Liaoning, China) was coated on a silicon pallet (Ultrapak^®^ 100 mm, Entegris, Billerica, MA, USA), and an SU-8 mold was obtained on the silicon pallet after the steps of pre-baking, exposure, post-baking, and development, as shown in Figure 1(ai–aiv). Then, as shown in Figure 1(av–aix), the PDMS was poured on the mold and coated using a spin coaster (Jiangsu Lebo Science, EZ4, Jiangyin, Jiangsu, China) at 500 r/min for 2 min and then heated at 65 °C for 150 min. After that, thin PDMS substrate was peeled off the mold. Afterward, a hole punch was used to punch 0.5 mm diameter holes at both ends of the channels (as shown in Figure 1(aix), injecting points). Then the PDMS structure was connected to another blank PDMS film by plasma bonding using a plasma cleaner (Tangshan Yanzhao Technology, YZD08-2C, Tangshan, Hebei, China). Finally, EGaIn was injected into the channels directly using a syringe. 

Figure 1b shows a three-dimensional rendering of the LM-based electrode and displays the structure of the implanted area in detail. The width and height of the LM channels are 100 μm and 80 μm, respectively, and the channels’ spacing in the implantation area is 100 μm. The 20 recording points were fabricated in a 6 mm × 7 mm area. Pt columns were inserted in the 20 recording points with an industrial microscope (Guangzhou Qifeng Technology Development Co., Ltd., 7-1200, Guangzhou, Guangdong, China). Another 20 endpoints were spaced 1.27 mm apart, fitting with the female pin header that is the computer’s connector. The female pin header (Shenzhen Zhongxin Electronic Technology, 20p, 1.27 mm, Shenzhen, Guangdong, China) was inserted in the 20 endpoints. The Pt columns and the female pin header were fixed in place with glue (Kafuter K-705).

### 2.3. Characterization of the LM-Based Electrode

The change in resistance of the electrode during bending was tested by the two-wire method, and the Agilent 34420A Meter (Agilent Technology, Beijing, China) was used to obtain data, with a recording rate of 10 data points per second. The bending frequency was about 12 s per time.

The electrochemical properties of encapsulated LM-based electrodes were tested using cyclic voltammetry (CV) and electrochemical impedance spectroscopy (EIS) on an electrochemical workstation (CHI660E Electrochemical Workstation, CH Instruments Inc., Shanghai, China). One LM-based electrode channel was used as the working electrode, and a Pt column with a diameter of 0.5 mm was used as the counter electrode (only the cross section was in contact with the electrolytic cell). The CV experiments tested the potential between −0.6 V and 0.8 V with a scan rate of 0.02 V/s. The EIS experiments applied a 10 mV sine wave with a frequency from 0.1 to 100,000 Hz. In addition, a length of 0.2 mm of 0.5 mm diameter platinum wire was used as a working electrode for comparison. CHI660E (CH Instruments Inc., Shanghai, China) was used to extract the parameters of the equivalent circuit.

### 2.4. Electrode Implantation Procedure

**Figure 2:**
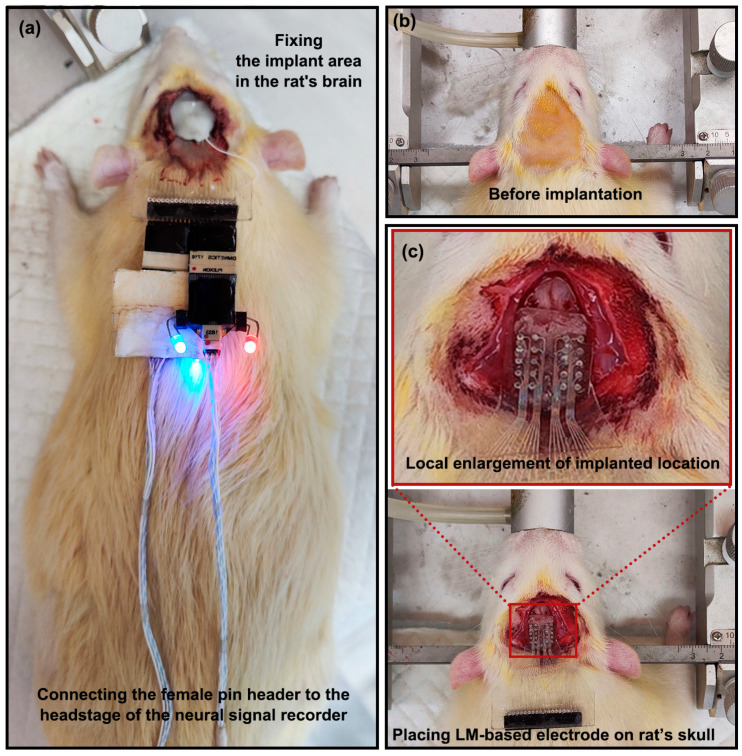
(**a**) Fixing the implant area in the rat’s brain and connecting the female pin header to the headstage of the neural signal recorder; (**b**) shaving the rat’s hair on the head before implantation and disinfecting the rat’s head with tincture of iodine; (**c**) placing the LM-based electrode on the rat’s skull and local enlargement of implant location.

As shown in Figure 2b, the head of the rat was first sterilized, and the sterilized area was slightly larger than the implanted area (extending 0.5 cm outward). Then the LM-based electrode was implanted into the rat. After that, the implantation area was fixed to the rat’s skull with dental cement, and the female needle was connected to the head stage of the nerve signal recorder, as shown in Figure 2a. The LM-based electrodes were in direct contact with the rat’s skull and performed signal acquisition. (The batch of rats, the process of implantation, and the process of signal acquisition were kept consistent).

All organizational and country-specific protocols for treating and caring for experimental animals were complied with. The Medical Ethics and Welfare approved all the laboratory procedures of the Beijing University of Technology Laboratory Animals Committee.

Animal experiments were carried out on a healthy and mature Wistar rat weighing approximately 250 g. The rat was anesthetized with isoflurane (3–4%) at a rate of 1 L/min and stabilized in a stereotactic frame during the implantation procedure. During the procedure, the rat was treated with an ointment to moisten its eyes. A heating pad was used to keep body temperature at 37 °C. Before implantation, a tincture of iodine was used to disinfect the rat’s head, and the hair on the head of the mouse was shaved, as shown in Figure 2b. An excision of approximately 2 cm was formed in the middle of the skull and widened by a small clamp. A small pair of tweezers was used to scrap the periosteum. The LM-based electrode was placed on the skull and pressed down to attach it to the skull, as shown in Figure 2c. An appropriate amount of dental cement was then used to fix the LM-based electrode, as shown in Figure 2c. The LM-based electrode, fixed with dental cement, did not move during signal acquisition. This ensured that a stable signal with a high dry ratio was recorded.

### 2.5. EEG Signal Recording and Cortical Source Localization

EEG signals through the LM-based electrode’s 20 channels were recorded and monitored by OmniPlex (Plexon Inc. Dallas, TX, USA). EEG signals were analyzed by open-source MATLAB-based tools, including OpenMEEG [35,36], FieldTrip [37], and sLORETA [38]. The obtained signals were pre-processed with automated algorithms and manual filtering to remove artifacts, including blinking, eye movements, respiration, heartbeat, and other muscle action processes. Signal space projection (SSP) was used to identify any repeatable artifacts by distinguishing their characteristic topographical patterns and removing the contribution to the EEG signals. The EEG recordings were then band-pass filtered with a cut-off frequency band of 0.1–60 Hz to prevent noise in the recordings. In addition, channels that were affected by noise were filtered out. 

The rat was sound-stimulated using sound pulses (1 ms, 2 kHz) a total of 38 times. The EEG signals were recorded during the stimulation sessions to analyze the current source of cortical responses evoked by auditory stimuli. As described previously, the cortical source localization technique was used to locate the stimulation-activated brain region [29].

## 3. Results and Discussion

### 3.1. The Flexible Characteristics of the LM-Based Electrode

**Figure 3:**
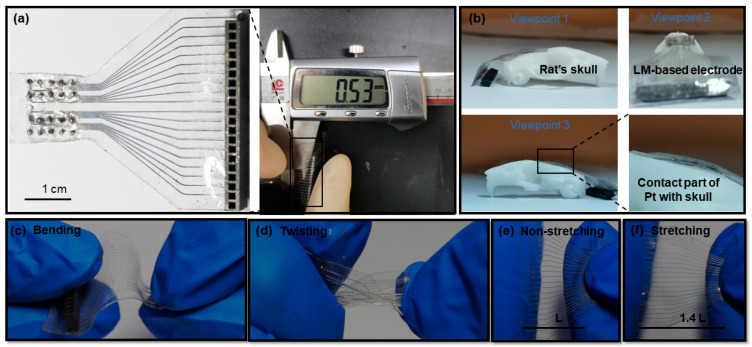
(**a**) The overall structure of the LM-based electrode (left) and the electrode thickness measurement (right); (**b**) three different views of the LM-based electrode forming conformal contact with the rat skull model and enlarged views; (**c**) bending of the intermediate connection part of the LM-based electrode; (**d**) twisting of the intermediate connection part of the LM-based electrode (180°); (**e**) non-stretching of the LM-based electrode; (**f**) stretching of the middle part of the LM-based electrode to 140%.

PDMS was chosen as the substrate material, which has been widely used as a soft and flexible substrate material in the past decades [39]. The low Young’s modulus of PDMS allows prepared soft devices to be deformed [33]. EGaIn was chosen as the conductive material for the 20-channel neural electrode array. EGaIn, as a “liquid”, has low viscosity (1.99 × 10^−3^ Pa∙s), high surface tension (0.624 N/m), and high conductivity (3.46 × 106 S/m) [40]. Therefore, the combination of LM-based electrodes with soft materials (PDMS) had excellent mechanical and electrical properties. The encapsulated complete electrode is shown on the left side of Figure 3a, and the thickness measurement of the electrode is shown on the right side of Figure 3a. The total length of the LM-based electrode was about 3.5 cm, and the thickness of the electrode was 0.53 mm (this thickness was used for subsequent experiments). The thickness of such electrodes prepared by microfluidic methods is in the hundreds of microns, while the thinnest thickness we could achieve was 0.18 mm. However, extremely thin electrodes suffer from poor encapsulation, which affects the signal acquisition. If the LM-based electrode is thick, it not only affects the comfort of the animal after implantation, but also reduces the flexibility of the whole electrode. Therefore, future implantable neural electrodes are expected to be developed toward thinness and lightness. The encapsulation technology of such electrodes is also crucial. The rat’s skull was made by a 3D printing machine (Suzhou Rayshape, shape1 HD, Suzhou, Jiangsu, China). Figure 3c–f show the bending, twisting, and stretching performance of the LM-based electrode.

As shown in Figure 3b, the LM-based electrodes were well adapted to the rat’s skull module when the electrodes were placed on the rat’s skull. The conductive material inside the LM-based electrode channel is liquid, and the substrate material is also a flexible polymer. We have demonstrated this property from three different perspectives. The part of the platinum column in contact with the rat skull was also amplified. In addition, the electrode could be easily bent, as shown in Figure 3c. Moreover, as shown in Figure 3d, this electrode could be twisted over 180°. Therefore, compared with rigid electrodes, LM-based flexible electrodes cause less damage to the body during the implantation process. Figure 3e,f show that the electrode could be stretched to 140%. This shows that LM-based electrodes possess mechanical properties that are closer to those of living organisms. Moreover, the outstanding adhesion to the skull enabled the LM-based electrode to obtain stable and clear signals.

### 3.2. The Resistance and Electrochemical Characteristics of the LM-Based Electrode

**Figure 4:**
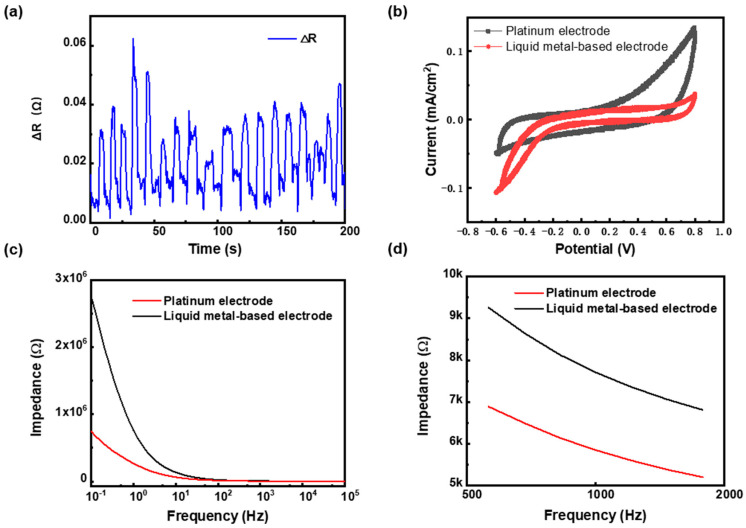
(**a**) The change in resistance of the electrode during manual bending; (**b**) the CV curve of the LM-based electrode and Pt electrode from −0.6 V to 0.8 V at a scan rate of 0.02 V/s (in saline); (**c**) the EIS curves of the LM-based and Pt electrodes under 0.1 up to 10^5^ Hz at a scan rate of 0.1 V/s (in saline, 10 mV sine wave); (**d**) the EIS curves of the LM-based and Pt electrodes under 500 up to 2000 Hz at a scan rate of 0.1 V/s (in saline, 10 mV sine wave).

When the electrode was implanted into the rat, the implanted area of the electrode was bent at the boundary due to pressing, as shown in the local enlargement in Figure 2c. To analyze the effect of bending, the electrode was manually bent; the change in the resistance of the electrode is shown in Figure 4a. The resistance of the electrode was 2.878 Ω, while the resistance changes during the bending process were no more than 0.06 Ω (less than 2%). This result fully ensured the stability of the electrode during implanting. At the end of implantation, neither the electrodes nor the rats moved. Therefore, the bending during implantation did not affect the signal acquired by the LM-based electrodes.

CV was used to assess the electrochemical response at the electrode/electrolyte interface, because the smoothness and flatness of the CV curve could reflect the stability of the electrode. The CV test results of the LM-based electrode and Pt electrode are shown in Figure 4b. It can be seen that both the LM-based and Pt electrodes’ CV curves were smooth, without apparent electrochemical reaction peaks, which indicates that both the LM-based electrode and the commonly used implanted Pt electrode had high stability.

The EIS test evaluated the interfacial impedance between the electrode and the electrolyte. The lower the interfacial impedance, the more precise the collected nerve signal and the lower the noise. The EIS test results for the LM-based electrode and Pt electrode are shown in Figure 4c,d. In the test frequency interval of 0.1–10^5^ Hz, as shown in Figure 4c, the maximum impedance of LM-based and Pt electrodes was 2.77 MΩ and 0.75 MΩ, respectively. The minimum impedance of LM-based and Pt electrodes was 5.2 KΩ and 4.1 KΩ, respectively. The impedance between the LM-based electrode and the saline was higher than that of the Pt electrode. However, both of them were within the same order. As shown in Figure 4d, the impedance of the LM-based electrode was 7.7 KΩ at 1 kHz, slightly higher than 5.9 KΩ for the Pt electrode, which was below 10 MΩ to enable a clear neural signal [38]. The excellent impedance performance was the basis for recording high-quality signals in rats. 

### 3.3. Stimulation and Cortical Source Localization after Implantation

**Figure 5:**
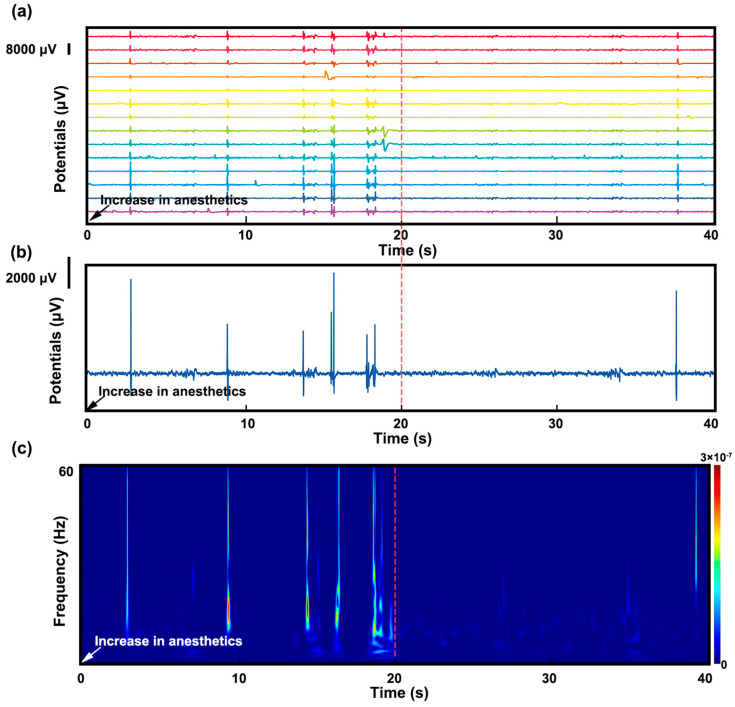
(**a**) A 40 s sample of raw EEG signals in the time domain of the rat under different ane−thetic doses; (**b**) one enlarged channel of EEG signals showing the details of the burst−suppression patterns; (**c**) the frequency spectrum of this EEG signal sample.

After electrode implantation, the EEG signal of the rat under different anesthetic doses was monitored. Figure 5a demonstrates a 40 s sample of these EEG signals in time domain. Two noisy channels in a total of sixteen channels were removed according to signal pre-process criteria. One of the channels is enlarged in Figure 5b to show the details of the potential change. Figure 5c shows the frequency spectrum of this EEG signal sample. 

The EEG signal showed burst-suppression patterns, characterized by intermittent high-voltage bursts alternating with isoelectric periods. This EEG pattern has often been identified during anesthesia, coma, hypothermia, and infantile encephalopathy. The RMS noise in isoelectric periods was about 100 μV, and the burst peak was up to 8000 μV. The signal-to-noise ratio was 80:1, which indicated that the quality of the collected signal was excellent.

The anesthetic dose was increased from 1 L/min to 3 L/min at the time 0 s, as shown in Figure 5. A decrease in the burst-suppression ratio was observed after 20 s, as shown in Figure 5a,b. The frequency spectrum in Figure 5c also revealed a low-level state of burst suppression after the anesthetic dose was increased. The change of burst-suppression ratio reflects the level of brain activity and the depth of anesthesia.

**Figure 6:**
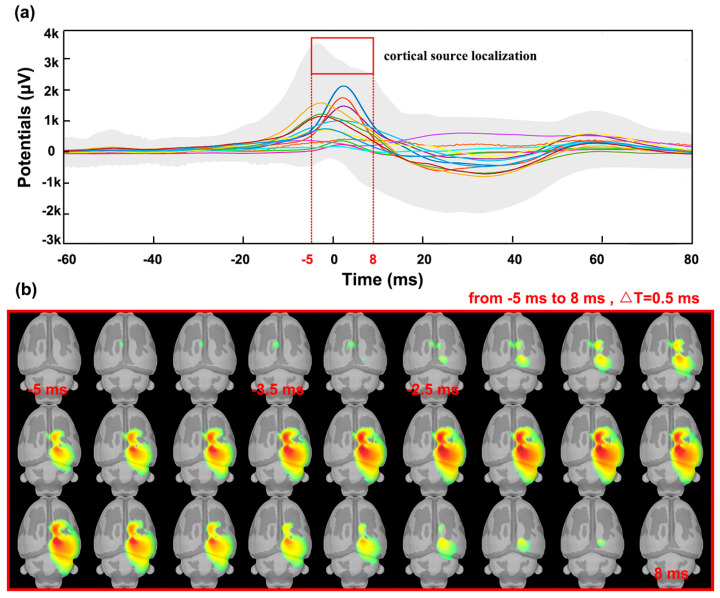
(**a**) Sound pulses evoked auditory−event−related potentials; (**b**) the current source density map showing auditory-stimulation-activated cortical areas.

Sound pulses stimulated the rats and triggered auditory-event-related potentials in the cerebral cortex. The corresponding cortical source potentials and activated brain areas were also obtained using source localization technique. The data were first segmented according to the peak through the EEG signal, and the waveform analysis of the obtained individual segments was performed to obtain the auditory-event-evoked potentials generated in the rat cerebral cortex. Figure 6a shows the characteristics of the auditory event-related-potentials, using colored lines to represent the evoked potentials of the individual channels and gray areas to represent the average potentials of all epochs. Starting at −20 ms, the average potential gradually increased; at −3.5 ms, a maximum of 3500 mV was reached. Around this time, the current source of the potential appeared on the left side of the brain, as shown in Figure 6b. At −2.5 ms, the current source spread to the right side. The activated cortical area on the right side then increased and disappeared completely at 8 ms. Subsequently, the averaged potential disappears at 50 ms.

## 4. Conclusions

In this paper, an LM-based 20-channel neural electrode array was developed using microfabrication technology. The deformation performance and the electrical properties of the electrodes were tested. The LM-based electrode demonstrated excellent deformability to be bent, twisted, and stretched, and it could form conformal contact with the skull surface of rats. Moreover, the electrical performance characterization proved that the LM-based electrode conductivity was slightly affected by bending, and its conductivity was comparable with that of platinum electrodes in the CV and EIS tests. This electrode could achieve high signal-to-noise-ratio EEG signal acquisition, and the anesthesia state of the rats was reflected in the burst-suppression patterns of the EEG signals. The auditory-activated cortical area was successfully traced by source localization technique. The LM-based electrode has been preliminarily proven to be useful as a neural information transmitter in a brain–computer interface system or as a probe to diagnose and treat neurological diseases.

## 5. The Future Perspectives

The advent of LM-based electrodes brings new opportunities and challenges for neuroscience research and neuro-engineering technologies. LMs have many advantages over conventional electrode materials: LMs possess excellent flexibility and plasticity, which allow them to adapt to different shapes and sizes of brain structures, so as to improve the acquisition efficiency and accuracy of EEG signals. LMs have extremely high electrical conductivity, which allow them to transmit EEG signals effectively, reduce signal attenuation and distortion, and improve signal clarity and stability. In addition, LMs will not produce adverse reactions from or damage to the human body and can be safely applied to EEG signal acquisition as well as recording for a long time. However, LMs with flow characteristics need to be encapsulated when made into an electrode. Therefore, future research on the encapsulation of LMs is essential. The choice of encapsulation material is the focus of attention. An encapsulation material that possesses biocompatibility, flexibility, and electrical conductivity is expected. In summary, LMs have extraordinary advantages as brain electrode materials that can improve the efficiency, accuracy, and stability of EEG signal acquisition and provide new possibilities for brain science research and clinical applications. LM-based electrodes have broad application prospects in the field of brain neural signal acquisition and are expected to become an important tool for brain science research and neurological disease treatment in the future.

## Data Availability

Data available on request from the authors.
